# The Prevalence of Precancerous Cervical Cancer Lesion among HIV-Infected Women in Southern Ethiopia: A Cross-Sectional Study

**DOI:** 10.1371/journal.pone.0084519

**Published:** 2013-12-20

**Authors:** Abel Gedefaw, Ayalew Astatkie, Gizachew Assefa Tessema

**Affiliations:** 1 Department of Gynecology and Obstetrics, College of Medicine and Health Sciences, Hawassa University, Hawassa, Ethiopia; 2 School of Public and Environmental Health, College of Medicine and Health Sciences, Hawassa University, Hawassa, Ethiopia; 3 Department of Reproductive Health, Institute of Public Health, University of Gondar, Gondar, Ethiopia; University of Buea, Cameroon

## Abstract

**Introduction:**

The magnitude of precancerous cervical cancer lesions as well as invasive cervical cancer is higher in HIV-infected women than non HIV-infected women. Thus, screening targeting HIV-infected women is being undertaken in developing countries, including Ethiopia. However, data on the prevalence and determinants of precancerous cervical cancer lesion among HIV-infected women in southern Ethiopia is lacking. Thus, this study aimed to assess the prevalence of and factors associated with precancerous cervical cancer lesion among HIV- infected women in southern Ethiopia.

**Methods:**

A hospital-based cross-sectional study was conducted from October 2012 to February 2013 among HIV-infected women in Southern Ethiopia. Four hundred forty eight HIV-infected women who had been screened and treated for precancerous cervical cancer lesion were included in the study. Data were collected by using structured and pretested questionnaire. Visual inspection with acetic acid was applied for screening and treatment. SPSS version 16.0 was used for data entry and analysis. Logistic regression analysis was fitted and odds ratios with 95% Confidence intervals and p-values were computed to identify factors associated with precancerous cervical cancer lesion.

**Results:**

Out of 448 study participants, 99 (22.1%) were found to be positive for precancerous cervical cancer. Being currently on highly active antiretroviral treatment (AOR=0.52, 95%CI: 0.35, 0.92), history of sexually transmitted disease (AOR=2.30, 95%CI: 1.23, 4.29) and having only one lifetime sexual partner (AOR=0.33, 95%CI: 0.20, 0.56) were factors associated with precancerous cervical cancer lesion.

**Conclusions:**

The prevalence of precancerous cervical cancer lesion among HIV-infected women in southern Ethiopia was found to be high. Intervention to access all HIV-infected women like scaling up the limited services and awareness creation should be undertaken. Measures aimed at preventing the acquisition and transmission of sexually transmitted diseases and reducing the number of sexual partners are required. Besides, early initiation of highly active antiretroviral treatment is important.

## Introduction

According to 2008 estimates, invasive cervical cancer (ICC) is the third most common cancer in women worldwide. Although cervical cancer is a preventable disease, it remains a leading cause of death among women in developing countries [1]. Globally, cervical cancer has a yearly incidence rate of 371,000 cases and an annual death rate of 190,000 with 80% of the cervical cancer mortalities occuring in developing countries. The problem is particularly severe in sub- Saharan Africa, where the age standardized incidence rate is 45 per 100,000 women [2,3].

According to the 2009 World Health Organization (WHO) report, the age-adjusted incidence rate of cervical cancer in Ethiopia is 35.9 per 100,000 women [4]. Despite this fact, very few women receive screening services [5]. Although there is no national cancer registry, reports from retrospective reviews of biopsy results have shown that cervical cancer, followed by breast cancer, is the most prevalent cancer among women in the country [6].

Since the onset of the Human Immunodeficiency Virus (HIV) epidemic, the United States Centers for Disease Control and Prevention (CDC) has classified cervical cancer as an Acquired Immunodeficiency syndrome (AIDS) defining cancer because of its close association with HIV infection [7]. Numerous studies have shown that the prevalence and incidence of cytologically detected squamous intraepithelial lesion (SIL) and infection of the cervix with human papilomvirus (HPV) are significantly more common in HIV-infected than non HIV-infected women [8-10]. Moreover, unlike other AIDS-defining cancers, the incidence of cervical cancer has not decreased substantially with the increasing use of antiretroviral therapy [11,12]. Hence, the development of a rational approach to the screening and the subsequent management of cervical disease in this segment of population are important.

In developed countries 75% of women are screened for cervical cancer, typically by Papanicolaou (Pap) smears and more recently HPV test, compared with 5% in developing countries [13]. The cytology based cervical screening has curbed the incidence of cervical cancer for decades [14]. Previous experiences in many sub-Saharan countries highlighted a lack of reliability of cytology based cervical screening at the population level due to lack of the necessary resources, infrastructure and technological expertise, together with the need for repeated screening at regular intervals [15,16]. 

The development of a rapid and affordable test for HPV, known identified causes of cervical cancer [17,18], makes a viable alternative to cytologic screening [19,20]. Presently, the two assays most widely used for the detection of genital types are polymerase chain reaction (PCR) with generic primers and the Hybrid Capture 2 (hc2) assay (Digene Corporation, Gaithersburg, MD, USA). Currently both of them are expensive, time-consuming, requires sophisticated laboratory infrastructure and essentially a research tool, not suited to be applied as a mass screening test particularly in resource-limited settings. However, the new rapid test developed by Digene Corp which is a modification of the hc2 test requires only basic laboratory tools, can be set up easily in the field and can be easily taught to health workers. This test has shown good concordance with the previous test and has the maximum potential to be applied as a population screening tool and has been used for screening both in developed countries and in resource-limited settings alone or in combination with other methods [21]. Such kind of screening method however is nonexistent in Ethiopia.

Low cost cervical cancer screening procedures based on Visual Inspection of the cervix with Acetic acid (VIA) or Visual Inspection with Lugol’s Iodine (VILI) have been proposed and adapted to resource-limited settings for years [22,23]. Although VIA has lower sensitivity and specificity than HPV tests, it has shown an equal validity compared to cytology in resource-limited settings [24,25]. VIA is reported to have 80% sensitivity, 92% specificity, a 10% positive predictive value and a 99% negative predictive value [26]. In some resource -limited countries, including Ethiopia, donor funding from the President’s Emergency Plan for AIDS Relief (PEPFAR) and the Global Fund has provided the resources and infrastructure for VIA screening and treatment of HIV- infected women and is being implemented since the past few years [27].

HIV infection and cervical cancer are major public health problems facing women in Ethiopia. More than 500,000 women are estimated to be infected with HIV [28] and at risk of developing cervical cancer. Though screening of precancerous cervical cancer lesion for HIV-infected women has been started in limited centers in Ethiopia, data on the prevalence and factors associated with the lesion are lacking. Knowledge about prevalence and associated factors is needed to identify HIV-infected women who are more likely to develop precancerous cervical lesion and to plan appropriate screening and treatment strategies. The present study provides health planners and policymakers with useful information that could lead to prevention of cervical cancer mortality and morbidity in HIV-infected women.

## Methods

A hospital-based cross-sectional study was conducted in three hospitals of Southern Nations Nationalities and People Region (SNNPR) of Ethiopia from October 2012 to February 2013. According to the 2007 census report, SNNPR has a total population of 15,321,000, of which 50.3% were females [29]. The prevalence of HIV in the region was 1% according to the 2011 Ethiopian Demographic and Health Survey (EDHS) [28]. The three hospitals—Hawassa University Referral hospital, Yirga Alem Regional Hospital and Wolayita Sodo Hospital— were included in the study as they are the only screening and treament centers for precancerous cervical cancer lesion (PCCL) in HIV- infected women in southern Ethiopia. These hospitals are 275-350 kilometers south of Addis Ababa, the capital city of Ethiopia. Each hospital has antiretroviral treatment (ART) clinic which provide care and treatment for more than 2,500 HIV- infected patients.

The sample size was calculated using the software Epi Info version 7 for a single population proportion considering the following assumptions. As there was no previous study done in Ethiopia, the prevalence of PCCL among HIV-infected women was assumed to be 50%; with 95% confidence interval, 5% absolute precision, and 10 % non response rate, the total sample size was estimated to be 422. However, during the study period there were a total of 458 eligible HIV-infected women (18 years and older) who were screened and treated for PCCL at the three hospitals and hence all of them were included in the study. HIV- infected women who had a history of diagnosed cervical cancer and those who had total hysterectomy were excluded from the study. Data were collected using structured and pretested questionnaire through face-to-face interview. Patients’ ART follow up charts were also reviewed. Four trained female diploma nurses supervised by two health officers collected the data during the study period. 

### Data/measurement

Trained nurses working in the screening and treatment centers of the three hospitals conducted the screening procedure. Un-lubricated bivalve speculum would be inserted into the vagina and the cervix visualized using a halogen focus lamp to identify the Squamo-Columnar Junction (SCJ). After cleaning away any excess mucus using a cotton swab, a five percent acetic acid solution was applied to the cervix for visual inspection with acetic acid. Findings were visible one minute after application. Precancerous lesions were defined as being dense aceto-white lesions with well defined margins observed within the vicinity of the transformation zone originating from the SCJ, or if the whole cervix or cervical growth turned white. A suspicion of invasive cervical cancer was defined as any cervical ulcer or growth being observed. Results of VIA were classified as negative, positive, or suspicious for Invasive Cervical Cancer (ICC) according to the International Agency for Research on Cancer (IARC) training manual [30]. Whenever there was uncertainty of the screening result the nurses consulted a trained gynecologist and he/she would confirm the diagnosis. The data were entered and analyzed using SPSS version 16.0 statistical package. Data cleaning was performed by running simple frequency distributions, summary statistics and cross-tabulation to identify and correct out-of-range, missing and inconsistent values. Descriptive and summary statistics were used to describe the data in relation to relevant variables. Bivariate and multivariate analyses were performed to test associations. Variables having p value ≤ 0.2 in the bivariate analyses were entered into a multiple logistic regression model to control the confounding effect amongst the variables. Odds ratio with their 95% confidence intervals were computed to identify the presence and strength of association, and statistical significance was declared if p < 0.05.

#### Data quality control

Data quality assurances were made by providing three days training for the data collectors and supervisors. Pretest was undertaken on 30 HIV-infected women in a hospital that has a screening and treatment center in a different region of the country. Based on the pretest, appropriate modifications were made before the actual data collection. Each day, the collected data were checked for completeness, and consistency by the supervisors and necessary corrections and changes were made when necessary. Moreover, the fact that the nurses consulted a trained and experienced gynecologist to confirm the diagnosis whenever there were uncertainties in the screening result helped to minimize observer bias.

#### Ethical statement

The study was reviewed and approved by the Institutional Review Board (IRB) of College of Medicine and Health Sciences, Hawassa University. Communications with the three hospitals administration were made through a formal letter obtained from Hawassa University. The purpose and importance of the study was explained to the participants. Informed written consent was obtained from each participant and anonymity was maintained to ensure confidentiality. Participation was on voluntary basis and they could withdraw from the study at any time. Women with positive results of VIA would be treated immediately following the screening while suspicious invasive cervical cancer findings would be sent to gynecology clinic for bunch biopsy.

## Results

### Socio demographic characteristics of the study participants

A total of 458 study participants were included in the study. Questionnaires from 10 women were excluded from the analysis because of incompleteness. The final analysis was thus based on data obtained from 448 (97.9%) of the study participants. The mean age of the study participants was 33 years (standard deviation (SD) =6.7). Nearly two third (65.4%) of the respondents were between 30-39 years old. More than half (54.0%) of them were married. Majority of the respondents (79.2%) were urban dwellers while 203 (45.0%) of them had attended primary school. One hundred twenty five (46.2.0%) of them identified their religion as Orthodox Christian and more than a quarter (27.9%) were Wolayita in Ethnicity (Table 1). 

**Table 1:** Socio-demographic characteristics of study participants in the three screening centers, Southern Ethiopia 2013.

Variable	Frequency	Percentage	Mean ±SD
Age group(in years)			33±6.7
20-29	97	21.7	
30-39	293	65.4	
≥40	58	12.9	
Religion			
Orthodox Christian	207	46.2	
Muslim	56	12.5	
Protestant	185	41.3	
Marital status			
Single	44	9.8	
Married	242	54	
Divorced	86	19.2	
Widow	76	17	
Ethnicity			
Amhara	119	26.5	
Oromo	50	11.2	
Sidama	85	19	
Wolayita	125	27.9	
Gurage	69	15.4	
Residence			
Urban	355	79.2	
Rural	93	20.8	
Education			
No formal education	114	25.5	
Primary education	203	45.3	
Secondary or above education	131	29.2	
Occupation			
Employed (Governmental and non- Governmental)	109	24.3	
Self employed ( merchant)	174	38.8	
House wife	137	30.6	
Other *	28	6.3	

(n=448).

^*^ student, daily laborer, job seeker

### Reproductive health characteristics of the study participants

 The mean age of menarche of the study participants was 14.53(SD±1.27) years. While the mean age of first sexual intercourse was 17.3(SD=2.51) years. More than one third (39.1%) of the participants had their first sexual intercourse after the age of 18 years. Similarly, mean age at first marriage was 17.9(SD±3.31) years and mean age at first birth was 19.68(SD±3.10) years. Moreover, three hundred and four (67.9%) of them had more than one life time sexual partners. Three quarters (74.6%) of the participants had given birth at least once with mean parity of 2.9(SD±1.99). Nearly two thirds (67.9%) of the participants had no history of abortion and 256 (56.9%) were not using any form of contraceptive during the study period. Nearly a quarter (23.7%) of them had no history of pelvic infection and about one third (30.1%) of them had no history of sexually transmitted disease (STD). Moreover, one out of five (21%) of the participants had a partner with history of STD (Table 2).

**Table 2:** Reproductive health characteristics of study participants in the three screening centers, Southern Ethiopia 2013.

Variable	Frequency	Percentage	Mean ±SD	
Age of menarche			14.5±1.3	
<15	359	80.1		
≥15	89	19.9		
Age at first sexual intercourse			17.3(SD±2.51)	
<15	150	33.5		
15-17	123	27.5		
≥18	175	39.1		
Age at first marriage (n=430) *			17.9±3.3	
<15	97	22.6		
15-17	123	28.6		
≥18	210	48.8		
Age at first birth (n=378) **			19.7±3.1	
<15	15	3.8		
15-17	72	23.1		
≥18	291	73.1		
Life time number of sexual partners			3.0(SD±4.2)	
One	144	32.1		
Two or more	304	67.9		
Family history of cervical cancer				
Yes	17	3.8		
No	431	96.2		
Ever history of smoking				
Yes	17	3.8		
No	431	96.2		
Parity				2.9±1.3
0	54	12.1		
1-4	334	74.6		
≥5	60	13.4		
History of abortion				
Yes	145	32.4		
No	303	67.6		
Current contraceptive use				
Yes	193	43.1		
No	255	56.9		
Ever history of pelvic infection				
Yes	106	23.7		
No	342	76.3		
Ever history of STD				
Yes	135	30.1		
No	313	69.9		
Ever history of STD in sexual partners				
Yes	94	21		
No	354	79		
Ever history of ulcerative genital lesion				
Yes	76	17		
No	372	83		

(n=448). *among those married. **among those who gave birth

### Immunological, behavioral, and other characteristics of the study participants

More than two-third of the study participants (67.9%) were currently on highly active antiretroviral treatment (HAART). The mean base line CD4 count of the participants while initiating HAART was 172.8 copies/mm^3^ (SD=85.6). The median duration of HAART use among those who were using HAART was 36 months (Inter Quartile Range (IQR) =18-60 months). Only 17(3.8%) of the study participants had ever history of cigarette smoking and similar proportion (3.8%) of them had family history of cervical cancer (Table 3)

**Table 3:** Immunological, behavioral and other characteristics of the study participants, in three screening centers, Southern Ethiopia, 2013 (n=448).

Variable	Frequency	Percentage	Mean ±SD, Median
Current use of HAART			
Yes	304	67.9	
No	144	32.1	
Base line CD4 count before HAART (n=304)*			172.8(SD±85.6), median=170
<200	206	67.9	
≥200	98	32.1	
Recent CD4 count after HAART(n=304)*			422.4(SD±237.4). median=400
<200	56	18.4	
≥200	249	81.6	
ART clinic follow up duration			
1-24 months	144	32.1	
25-48 months	125	27.9	
>48 months	179	40	
Current HAART use duration(n=304) *			Median-36 months (IQR: 18-60)
1-24 months	118	38.8	
25-48 months	94	30.9	
>48 months	92	30.3	
Age at first sexual intercourse			17.3(SD±2.51)
<15	150	33.5	
15-17	123	27.5	
≥18	175	39.1	
Life time number of sexual partners			3.0(SD±4.2)
1	144	32.1	
≥2	304	67.9	
Family history of cervical cancer			
Yes	17	3.8	
No	431	96.2	
Ever history of smoking			
Yes	17	3.8	
No	431	96.2	

^*^ among those who were on HAART

### Prevalence of precancerous cervical cancer lesion among HIV-infected women

Out of 448 screened HIV- infected women, 99 (22.1 %) [95% CI: 18.3%-25.9%] were found to be positive for PCCL. Three hundred forty five (77%) of them were negative for precancerous cervical cancer and four (0.9%) of them were suspicious for invasive cervical cancer and sent to gynecologic clinic for appropriate biopsy for confirmation of invasive cervical cancer. 

### Factors associated with precancerous cervical cancer lesion among HIV-infected women

Women with positive results of the precancerous cervical cancer screening and those of suspicious invasive cervical cancer were grouped together for logistic regression analysis as the former is the predecessor of the same disease entity, however, cognizant that a positive VIA result does not necessarily equates with a cervical cancer diagnosis.

Both bivariate and multivariate logistic regression analyses were run to determine factors associated with precancerous cervical cancer lesion. It was found that age of the participant, educational status, occupation, parity, being currently on HAART, lifetime history of pelvic infection, sexually transmitted disease (STD), ulcerative genital lesion, age at first marriage, age at first sexual intercourse, and life time number of sexual partners were statistically significant during bivariate logistic regression analysis at P- value of ≤0.2. However, only lifetime history of STD, being currently on HAART and life time number of sexual partners had statistically significant association with precancerous cervical cancer lesion in the multivariate analysis at P-value of <0.05.

Participants who were currently on HAART were 48% less likely to have precancerous cervical cancer lesion than those who were not on HAART [AOR=0.52, 95%CI: 0.35, 0.92]. HIV-infected women who had one lifetime sexual partner were 67% less likely to develop precancerous cervical cancer lesion than those having more than one life time sexual partners [AOR=0.33, 95%CI: 0.20, 0.56]. Additionally those women who had ever history of STD were about 2.3 times more likely to have precancerous cervical cancer lesion than those without history of STD [AOR=2.30, 95%CI: 1.23, 4.29] (Table 4). 

**Table 4:** Logistic regression analysis of factors associated with precancerous cervical cancer lesion (PCCL) in Southern Ethiopia, 2013.

Variable	PCCL		COR(95%CI)	P-value	AOR(95%CI)	P-value
	yes	No				
Age				0.18		0.086
20-29	78	19	0.66(0.27-1.61)	0.19	1.37(0.49-3.81)	0.551
30-39	217	76	0.46(0.21-1.01)	0.05	0.67(0.28-1.60)	0.366
≥40	50	8	1		1	
Education				0.177		0.039
No formal education	97	17	1.41(0.72-2.76)	0.20	1.41(0.62-3.25)	0.414
Primary school	143	60	0.59(0.35-0.99)	0.05	0.62(0.33-1.17)	0.137
Secondary school and above	105	26	1		1	
Occupation				0.182		0.586
Employed	86	23	2.08(0.85-5.12)	0.11	1.01(0.66-6.08)	0.223
Self employed	134	40	1.86(0.78-4.35)	0.15	1.97(0.70-5.49)	0.198
House wife	107	30	1.98(0.83-4.74)	0.13	2.02(0.72-5.64)	0.181
Others*	18	10	1		1	
Current HAART use				0.07		0.025
Yes	224	80	0.53(0.32-0.89)	0.02	**0.52(0.35-0.92)**	**0.015**
No	121	23	1		1	
Parity				0.119		0.088
0	31	13	0.38(0.15-0.94)	0.04	0.42(0.12-1.148)	0.084
1-4	245	79	0.49(0.25-0.98)	0.04	0.54(0.22-1.29)	0.165
≥5	69	11	1		1	
Ever history of pelvic infection				0.023		0.082
Yes	92	14	2.31(1.25-4.26)	0.007	1.84(0.94-3.58)	0.075
No	253	89	1		1	
Lifetime history of STD				0.00		0.013
Yes	118	17	2.63(1.49-4.63)	0.001	**2.30(1.23-4.29)**	**0.009**
No	227	86	1		1	
Ever history of genital ulceration				0.03		0.178
Yes	65	11	1.94(0.98-3.84)	0.06	1.65(0.79-3.45)	0.186
No	280	92	1		1	
Age at first sexual intercourse				0.121		0.362
<15	114	36	0.73(0.43-1.23)	0.16	0.96(0.44-2.10)	0.918
15-17	86	37	1.53(0.89-2.63)	0.13	1.60(0.72-3.54)	0.249
≥18	145	30	1		1	
Age at first marriage				0.200		0.392
<15	78	19	1.15(0.63-2.10)	0.19	1.64(0.68-3.95)	0.267
15-17	88	35	0.71(0.42-1.12)	0.18	0.89(0.44-1.80)	0.743
≥18	164	46	1		1	
Life time number of sexual partner				0.023		0.001
1	93	51	0.38(0.24-0.59)	0.000	**0.33(0.20-0.56)**	**0.000**
≥2	252	52	1		1	

^*^ student, daily laborer, job seeker. COR: Rrude Odd Ratio. AOR: Adjusted Odd Ratio.

## Discussion

Cervical cancer is the most common cancer in women in sub-Saharan Africa and second to breast cancer in northern Africa. In sub-Saharan Africa, it accounts for 22.2% of all cancers in women and it is also the most common cause of cancer-related death among women [1]. HIV-infected women are at a higher risk of developing precancerous as well as invasive cervical cancer lesion than non HIV-infected women [8,9]. Knowing the prevalence and associated factors of precancerous cervical cancer lesion among HIV-infected women helps to take preventive measures and to know the screening requirements.

In this study the prevalence of precancerous cervical cancer lesion among HIV-infected women was found to be 22.1% (95% CI: 18.3-25.9) which is comparable with a previous report of sub-Saharan African countries [31]. This high prevalence of precancerous cervical cancer lesion reveals that cervical cancer is a significant public health problem among HIV-infected women though the prevalence of precancerous cervical cancer in HIV-uninfected women is unknown in the study area. Despite the high prevalence of the lesion in southern Ethiopia, only three hospitals were providing the screening and treatment service which significantly hampered the service accessibility to all HIV-infected women in the region. This could be because of the limited resources available for treatment of positive precancerous cervical cancer lesion after screening with VIA as the current limited available services are donor dependant [27]. 

Higher prevalence of precancerous cervical cancer among HIV infected women than shown by this study has been reported in South Africa (66.3 %), Uganda (73%), and Zambia (76%) [32-34]. Studies done in Kenya and Rwanda also found the prevalence of precancerous cervical cancer to be 26.7% and 24.3% respectively, which is comparable to the result of the current study [35,36]. A lower prevalence of precancerous cervical cancer than the current study has also been reported from studies done in Botswana, Côte d'Ivoire, and Nigeria. In Botswana, a cervical cancer screening programme of 2,175 HIV-positive women based on VIA found that the proportion of precancerous cervical cancer lesion confirmed by histology was 15.2% [37]. Studies recently conducted in Côte d'Ivoire and Nigeria found the prevalence of precancerous cervical cancer lesion to be 11% and 6% respectively [38,39]. The differences among findings of prevalence of precancerous cervical cancer lesion in different regions of Africa could be partly due to differences in the sexual practices of the women studied [7]. Having multiple sexual partners because of cultural differences increases the risk of acquiring HPV, and in turn, the development of cervical pre-cancer and cancer. In Nigeria, where the lowest prevalence was reported, 96% of the study participants had two or less lifetime sexual partners [39]. In South Africa, one of the highest reported, the median number of sexual partners was four [32]. In the present study the mean number of lifetime sexual partner is three and might be one of the contributing factors for the high prevalence. Unlike the findings from South Africa and Nigeria [32,39], in Côte d'Ivoire [38], though 56% of women had an average of five lifetime sexual partners, prevalence was lower than the finding in the present study, making the association unlikely. It could also be a result of the limitation of self-reported sexual practices. The other possible explanation for the different prevalences could be the differences in the study populations. The highest prevalence, reported from Zambia, was based on only 150 women, all of whom were severely immune compromised [34]. The lowest prevalence reported from a Nigerian study, was based on only HIV-infected women who started ART (a known protective factor of precancerous and cervical cancer) [39].

The higher proportion of positive precancerous cervical cancer in the present study might be due to the population’s low level of awareness about the disease [40] and the insufficient attention given to cancer in the country as other infectious diseases like tuberculosis , malaria, and HIV overshadow it [41].

The present study found that HIV-infected women and those who were currently on HAART were 48% less likely to be positive for precancerous cervical cancer lesion than those not on HAART. First, this could occur because HAART prevents the development of precancerous and invasive cancer. Secondly, it will cause regression of the lesion [42]. This finding is consistent with a study conducted in Kenya [35]. This study also found that women who had one lifetime sexual partner were 67% less likely to develop precancerous cervical cancer lesion than those having more than one life time sexual partners. A study conducted in Tanzania is also in line with this finding [43]. The possible justification could be that as the number of sexual partners increases they become more prone to acquiring the HPV infection, which is the causative agent for precancerous cervical cancer and invasive cervical cancer [17,18]. Though data is not yet available on the HPV burden in Ethiopia; in Eastern Africa, where Ethiopia belongs to, about 33.6% of women are estimated to harbor cervical HPV infection at a given time. Moreover, in Ethiopia 90.2% of invasive cervical cancers are attributed to HPVstrain 16 or 18 [44].

Lifetime history of sexually transmitted disease was associated with precancerous cervical cancer lesion. Women who ever had a history of STD were about 2.3 times more likely to develop precancerous cervical lesion than those who had never had STD. This finding is consistent with a study done in Zimbabwe [45]. This could be due to the sexually transmitted nature of HPV infection [7]. Unlike studies done in Kenya [35], Nigeria [39] and Tanzania [43] which declared the association between precancerous cervical cancer lesion and CD4 count among HIV-infected women, the current study didn’t show any association between PCCL and CD4 count (base line CD4 count before HAART initiation, recent CD4 count after HAART use). This could be due to the significant difference in CD4 count while initiating HAART in the different studies. In the present study, majority (67.9%) of the participants were severely immunocompromised while initiating HAART with CD4 count of less than 200 while only 12.2% in Tanzania [43] and 45% in Kenya [35]. 

The present study included only HIV infected women voluntarily screened for precancerous cervical cancer and included only the three available screening centers in the region so that generalizability of findings to all HIV-infected women in southern Ethiopia may be limited. Moreover the inherent subjectivity of visual screening methods could affect the study’s findings in any direction (i.e., prevalence could overestimated or underestimated to some extent).

## Conclusions

In conclusion, the prevalence of precancerous cervical cancer lesion among HIV infected women in southern Ethiopia is high similar to the situation in other sub-Saharan African countries. Lifetime history of STD, having more than one life time sexual partners, and being currently on HAART were factors that were significantly associated with precancerous cervical cancer lesion. Scaling up the limited screening and treatment centers is required to increase access of all HIV-infected women to the service. Awareness creation on the availability of screening and treatment services is also necessary so that HIV-infected women could use the service. Besides, measures aimed at preventing the acquisition and transmission of sexually transmitted diseases and reducing the number of sexual partners are required. Early initiation of highly active antiretroviral treatment is also important.
